# Experimental study on the properties of modern blue clay brick for Kaifeng People's Conference Hall

**DOI:** 10.1038/s41598-021-00191-z

**Published:** 2021-10-19

**Authors:** Shaochun Ma, Youwen Wu, Peng Bao

**Affiliations:** 1 Kaifeng Research Center for Engineering Repair and Material Recycle, School of Civil Engineering and Architecture, Henan University, Kaifeng, 475004 Henan People’s Republic of China; 2 College of Water Conservancy & Engineering, Zhengzhou University, Zhengzhou, 450001 People’s Republic of China

**Keywords:** Civil engineering, Structural materials

## Abstract

This article presents building assessment research comprising on-site inspections, indoor scientific tests, and material performance studies on the wall blue clay bricks in the Kaifeng People’s Conference Hall, objectively developing an enhanced scientific understanding to renovate modern buildings. Using X-ray diffraction (XRD), scanning electron microscopy (SEM), alongside a parametric study of density, moisture content, water absorption, void ratio, frosting, compressive strength, and softening coefficient in assessing the material health of the blue clay bricks and it’s non-key parts, in developing “appropriate and compatible renovation” to repair contemporaneous buildings. The composition, pore characteristics, weathering degree, and mechanical properties of the blue clay brick samples were analyzed. These parameters showed that blue clay brick fired at less than 1000 °C; the main mineral composition as quartz, followed by albite, mica, and anorthite. Its density was 1.573 g/cm^3^, less than the 1.70 g/cm^3^ of ordinary clay brick. According to the standards, the water absorption was greater than that of regular sintered bricks by more than 18% and was slightly frosted. Compressive strength being less than MU10 did not meet the current design specifications for masonry. Its softening coefficient was between 0.70 and 0.85, but its water resistance was relatively good. The research results provide an essential reference for judging the health and longevity of modern buildings to achieve scientific guidelines for practical protection.

## Introduction

Ancient buildings in China were primarily wooden and masonry structures, which had poor fire resistance and durability. Nonetheless, masonry structures had better engineering adaptability, fire resistance, and durability. Most of the materials for ancient Chinese load-bearing building structures were blue bricks^1–4^, with a long history. Production and use of blue clay bricks commenced during the Warring States Period, but the technology lacked maturity at that time. In the Qin-Han dynasty, the firing process and key technologies of blue clay bricks and gray tiles reached a high level, but blue clay bricks were used only in the royal palace and important buildings. However, blue clay bricks were used more widely again during the Han Dynasty. During the Three Kingdoms, Wei and Jin Dynasties, blue clay bricks spread from the palace to city buildings. Finally, during the Ming and Qing Dynasties, the use of blue clay bricks was popularized^5^. Blue clay bricks were made of clay, with standard size specifications of 240 × 115 × 53 mm^3^, 60 × 240 × 10 mm^3^, 75 × 300 × 120 mm^3^, 100 × 400 × 120 mm^3^, 400 × 400 × 50 mm^3^, and so on. Thus, the blue brick witnessed changes in history, from paving the floor to building the wall.

The Opium Wars began in 1840 until the People’s Republic of China in 1949. The architecture of China in this period was thus called modern architecture. Construction was then in transition, connecting past with the next, intersecting China with the West, and the succession of the old to the new. Historic masonry structures were an essential part of China’s cultural heritage^6,7^. They played a vital role in the cultural inheritance, crystallized the working people’s wisdom in different periods, and had high artistic and scientific value^8^. However, historical changes fostered long-term erosion of the natural environment^9–11^, and human beings’ improper short-sighted use in reconstruction, the masonry structure of modern buildings suffered various degrees of damage^12–14^. For example, wall pulverization and peeling, wall cracking, large-scale frost on the wall, and decreased brick strength were unfavorable to the structure’s safety. Therefore, the state had successively issued many related policies to protect. For example, the “Notice of the State Council on Strengthening the Protection of Cultural Heritage” stated that historical and cultural heritage protection should be followed by “protection first, rescue first, rational use, and strengthened management”.

With the introduction of protection policies for ancient buildings, the protection of masonry brick structures had attracted increased attention from many people of varying walks of life. Many domestic and foreign scholars studied antique bricks’ fundamental properties and damage mitigation from different angles. Gao et al.^15^ analyzed the properties of various materials through (XRD), X-ray fluorescence (XRF), Fourier transformed infrared spectrometer (FTIR), Energy dispersive spectrometer (EDS) technology, and related physical tests on Xi’an city wall tiles and inscribed the words “Qianwei” as the seal. The mechanical properties of “Qianwei” bricks decreased slightly under environmental erosion, but their physical and chemical properties were better, reflecting the superior brick-making technology of the Ming Dynasty. Li et al.^16^ carried out the 24 h atmospheric water absorption testing, five-hour boiling water absorption, porosity, and composition analysis using XRD on 32 ancient bricks from the Ming and Qing Dynasties in Shanxi Province. And found that Shanxi’s old bricks’ 5-h boiling water absorption increased with increased porosity. Dynasties and regions had a particular influence on the composition of ancient bricks. To explore the main factors of the weathering of blue bricks in the ancient city of Pingyao, Liu et al.^17^ conducted various performance studies on the blue clay bricks in the old town of Pingyao. The results showed that the crystallization and freeze–thaw cycles of soluble salts were the two main factors leading to the weathering of these bricks. Kharfi et al.^18^ collected a terracotta brick from a famous Roman city and successfully determined its year and source of production materials through thermoluminescence (TL) technology and XRF. Bhattarai et al.^19^ used ASTM standards to study the physical and mechanical properties of seven ancient clay brick samples from the Kathmandu Valley of Nepal. They believed that the durability of old bricks was directly affected by physical properties such as water absorption, apparent porosity, and bulk density. Konior et al.^20^ considered that the building was made of basic materials (brick, plaster, wood, concrete, cement) through traditional techniques, and the size and degree of component damage affect the bearing capacity of the building. Maria Soledad et al.^21^ considered that due to the limited production technology of brick walls in historical buildings at that time, artificial masonry made the pores in the walls larger and easier to absorb water. Veronica et al.^22^ concluded that the existence of moisture would reduce the thermal and mechanical properties of wall blue bricks, and thus affecting the building bearing capacity. Wang et al.^23^ analyzed the chemical elements, crystal composition and microstructure of ancient blue bricks, and put forward that aluminum silicate content and sintering temperature were the main reasons that affect the difference of surface roughness and water absorption of blue bricks. Li et al.^24^ believed that in blue-brick masonry buildings, the rise of humidity would lead to deterioration and damage of blue-brick materials, and a series of hazards such as mildew would occur on the walls. Ding et al.^25^ carried out the compression test of blue brick masonry in modern cultural relic buildings. Based on the analysis of test data, the design value of compressive strength of blue brick masonry was determined. The stress–strain curve was established and the reference value of the elastic modulus for masonry was recommended. Cheraghcheshm et al.^26^ modified the brick surface with silver nanoparticles and characterized the modified brick nanocomposite by various technical means. The research showed that the application performance of the brick had been improved.

Based on the above research, it can be seen that many scholars' research on blue brick materials mainly include the water absorption, salt resistance and compressive properties of brick materials. They thought that the nature of ancient bricks varied significantly in different dynasties and different regions. Therefore, it proved challenging to achieve “compatible renovation” technique when making antique blue bricks to match the particular period according to different periods and regions. In view of the current research situation, the conservation of ancient buildings and cultural relics was a popular research direction. However, there were still some limitations in the research methods, research means, research depth and test instruments. In addition, when scientifically protecting ancient buildings, the protective materials and protection measures need to be selected according to the degradation mechanism of cultural relics and the properties of the materials^27–29^. Scientific study and protect the Kaifeng People’s Conference Hall and understanding the characteristics of its blue bricks’ structure and mechanical properties, we carried out a series of on-site testing and experimental research on the People’s Conference Hall wall blue clay bricks. Through the basic material tests, we gathered the needed basic properties. When repairing a building structure from this period, materials needed to be similar to maintain compatibility with the historical building materials and achieve “compatible renovation”^30^. Therefore, this article will provide a basis and reference for selecting protection materials, protection measures, and researching antique blue clay brick craftsmanship for historical buildings in this period.

Therefore, it was very necessary and meaningful to carry out material experimental research on modern and traditional architectural blue brick materials and scientifically evaluate their properties. By studying the material properties of modern bricks, the test method proposed could enrich or supplement the test scheme of ancient bricks. Through the basic material tests, we gathered the needed basic properties. When repairing a building structure from this period, materials needed to be similar to maintain compatibility with the historical building materials and achieve “compatible renovation”. It provided guiding suggestions for the formulation of protection schemes of modern and contemporary buildings and enriched the evaluation indexes of maintenance and reinforcement of modern buildings. It also laid a foundation for subsequent research on the influence of the surrounding environment on the performance of blue brick materials and provided theoretical support for the research on traditional building damage and health detection technology. The purpose of scientific protection and repair of ancient masonry structure was realized.

### Working condition introduction

**Figures 1–3:**
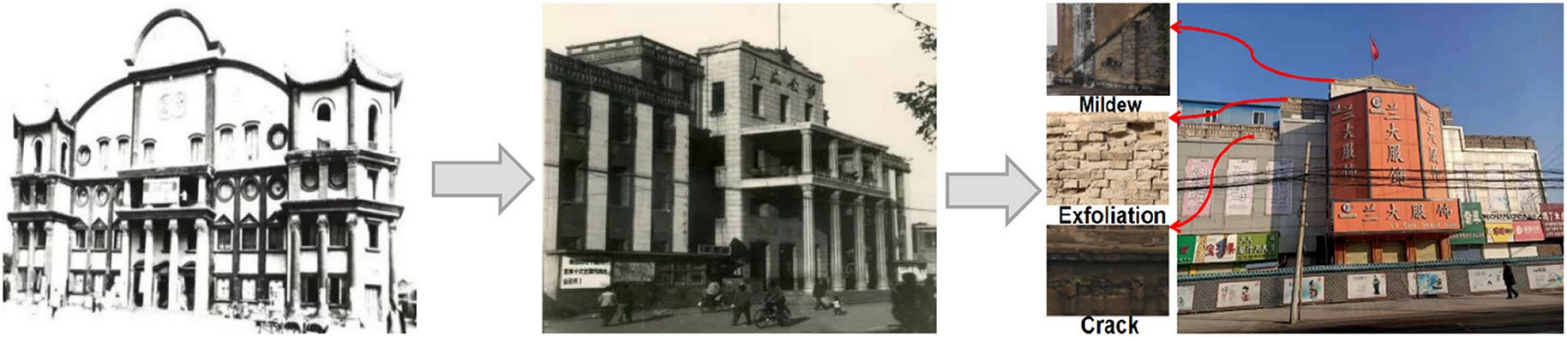
**Figure 1** Early completion of construction. **Figure 2** Transformation by posterity. **Figure 3** Expansion of shops outwards.

The Kaifeng People’s Conference Hall, built-in 1928 (nearly a 100 years ago), was the earliest large-scale modern theater in Kaifeng City and the earliest modern conference venue in Henan Province. In 2002, it was rated as a cultural relics protection unit in Kaifeng City. In the beginning, the People’s Conference Hall construction was built to promote social education, and its building materials were derived from the bricks, tiles, and wood removed from the temple. The brick and timber from the demolished Temple of Suffering were used in the construction, and the shape was a semi-circular roof, as shown in Fig. 1. After founding the People’s Republic of China, the People’s Conference Hall was a long-term venue for drama performances, and large conferences were also often held. Therefore, the government allocated funds to demolish the original dome roof and use the material removed to transform the shape into a pipe-cap chair-like facade, as shown in Fig. 2. Later, commercial shops leased it, and later generations expanded the original building partly based on the original structure to expand the limited space in the shopping mall, as shown in Fig. 3. The original walls of the People’s Conference Hall had been exposed long-time to natural environmental erosion withstanding wind, sun, rain, and snow. As a result, the blue bricks on the wall had flaked, powdered, and cracked. Part of the wall close to the ground showed a large area of chalk shedding. Although it had been repaired and reinforced several times, the deterioration still existed and was worsening. In the short term, these weathering phenomena had progressively cannibalized the founding material and the historical information embedded in the masonry brick structure. In the long term, quantitative changes caused qualitative changes, and the weathering of blue bricks on the wall inevitably led to the weakening and instability of the entire structure^31–34^, which was detrimental to the structure’s safety.

The long history of Kaifeng People’s Conference Hall harnessed the working peoples’ wisdom, witnessed many historical events, and acquired high artistic and scientific value. However, a series of damages and diseases had appeared on the People's Conference Hall’s original wall, threatening the structure’s overall stability and safety. Therefore, to better protect the People’s Conference Hall, the blue bricks on the wall’s fundamental nature were tested and analyzed. The blue brick’s mineral composition and pore characteristics were obtained through phase analysis and pore characteristics analysis. Preparing antique blue clay bricks of the same period provided the basis for selecting raw materials. The test results were compared with the corresponding indicators of standard sintered bricks through density, moisture content, and water absorption tests. Through comparison, the main reason for the weathering of blue bricks on the wall was found, which provided a theoretical basis for protecting the future of Kaifeng People’s Conference Hall. Through the void ratio determination, the pore characteristics inside the brick were more intuitively assessed. It can indirectly recall the weathering situation of the Kaifeng People’s Conference in the past 100 years to provide particular theoretical support for its maintenance and reinforcement. Through the frost test, the impact of the local conditions in Kaifeng on the blue bricks on the wall was evaluated, and it provided theoretical support for the future protection of the Kaifeng People’s Hall. The compressive strength of the bricks determined gave the basis for assessing the strength of the bricks. Through the softening coefficient test, the water resistance of the wall blue bricks was explored. The research results directly served the specific repair work of the People’s Conference Hall and provided a reference for the material properties of contemporaneous buildings.

Note: Fig. 1 and Fig. 2 are obtained from Kaifeng Archives. Figure 3 is obtained by the author personally on site.

## Material and instruments

### Preparation of the test samples

**Figures 4–6:**
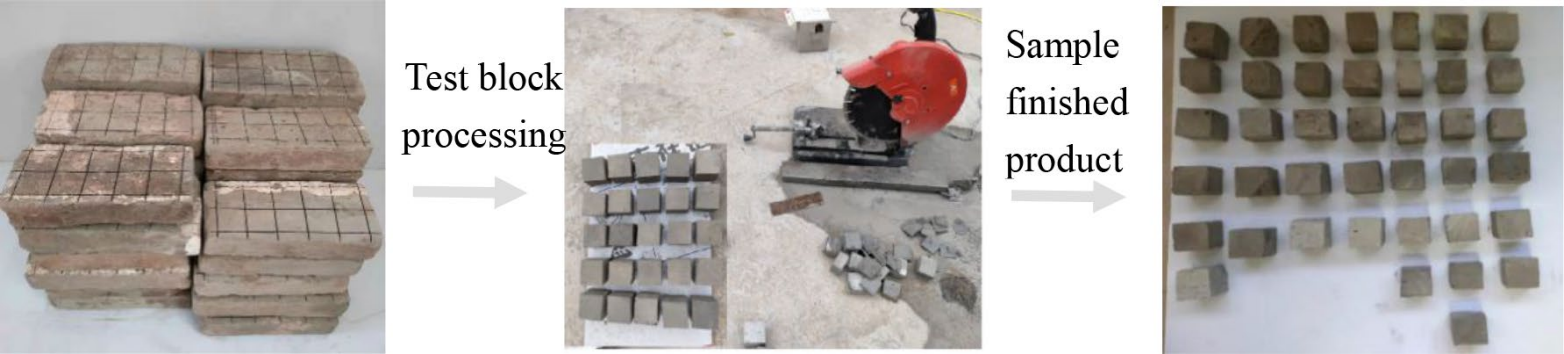
**Figure 4** Brick samples. **Figure 5** Specimen cutting and grinding. **Figure 6** Sample finished product.

The samples came from partially blue clay bricks on the walls of the Kaifeng People’s Conference Hall. Before performing various basic performance tests, the blue clay bricks were pretreated, and Fig. 4 shows some of these bricks. First, we removed the floating dust and mortar remaining on the brick surfaces, and then using a marker, drew a small grid of 50 mm × 50 mm on the cleaned surfaces. Then, following “Material for maintenance and conservation of historic architecture-Grey brick” (WW/T 0049-2014), the blue bricks were cut along the small grid drawn, using a cutting machine (see Fig. 5). Finally, cut and polished the blue bricks to make 50 mm × 50 mm × 50 mm cube samples (as seen in Fig. 6).

### Test instruments

The phase analysis of the samples was tested with an (XRD) instrument model D/max2400, the test voltage was 40 kV, the current was 35 mA, the scanning angle was 10°–90°, and the step size 0.02° (2θ), the scanning time per point was 0.15 s. Scanning electron microscopy was used to observe the microscopic morphology; the SEM model was JSM-7610 F. The test acceleration voltage was 5.0 kV, beam current was 80,100 Ma, the resolution was 1.0 nm/15 kV, sample magnifications 500×, 2000×, and 5000×. Sample density, moisture content, water absorption, frosting, compressive strength test, and softening coefficient test of the sample were tested according to the relevant procedures in GB/T 2542-2012 “Test Methods for Wall Bricks”^35^. The minimum graduation value of the balance used in the density, moisture content, and water absorption test was 0.01 g, and the blast dryer adopted was the 101-type blast drying box. For the hole ratio and frosting tests, we also needed to prepare graph paper and soaking containers. The mechanical performance test to determine the compressive strength and softening coefficient test used the WDW-600 E microcomputer-controlled electronic universal testing machine; the maximum load of the testing machine was 60t.

## Methodology

### Phase analysis

The mineralogical phase analysis of the clay-based brick was one of the widely accepted tools to approximately evaluate their firing temperature range, physical-sintering properties, and durability^36,37^. Hence, mineral composition analysis was of great significance in studying the physical properties of clay bricks.

The middle part of the sample was hammered and crushed to avoid the analysis results from being influenced by weather factors. Ground the crushed sample into a powder with an agate mortar. Used an XRD instrument to detect and obtain XRD patterns. We imported the obtained spectrum into the Jade analysis software to analyze the mineral composition of the ordinary sintered red brick and the blue clay blue brick. Finally, we combined drawing tools to generate a mineral composition diagram.

### Characteristics of pore

Took out a cut sample (about 5 mm square) from the central part to be the test sample. Dried the sample to a constant weight, then used conductive glue to paste the sample on the copper table and gold-coated the sample surface under vacuum. Finally, a JSM-7610 F (Field Emission Scanning Electron Microscope-FESEM) observed and imaged the microscopic morphology of the sample. FESEM settings used were; 50 kV acceleration voltage, 80,100 MA beam current, resolution of 1.0 nm/15 kV, sample magnifications of 500×, 2000×, and 5000×. The smooth sample surface, tightness of the pores between particles, and the particle structure within the sample were studied.

### Density, moisture content, and water absorption test

Density intuitively reflects the physical properties and the degree of compactness of a material. Moisture content and water absorption are vital parameters as they quantitatively indicate the quality of the clay brick and its ability to absorb and permeate water. Therefore, the testing gave the sample’s density, moisture content, and water absorption. Comparable results obtained from similar testing on standard sintered bricks served as indicators to ascertain the weathering state of blue clay bricks and formed a theoretical basis for protecting the future of Kaifeng People’s Conference Hall.

#### Experimental method

**Figure 7:**
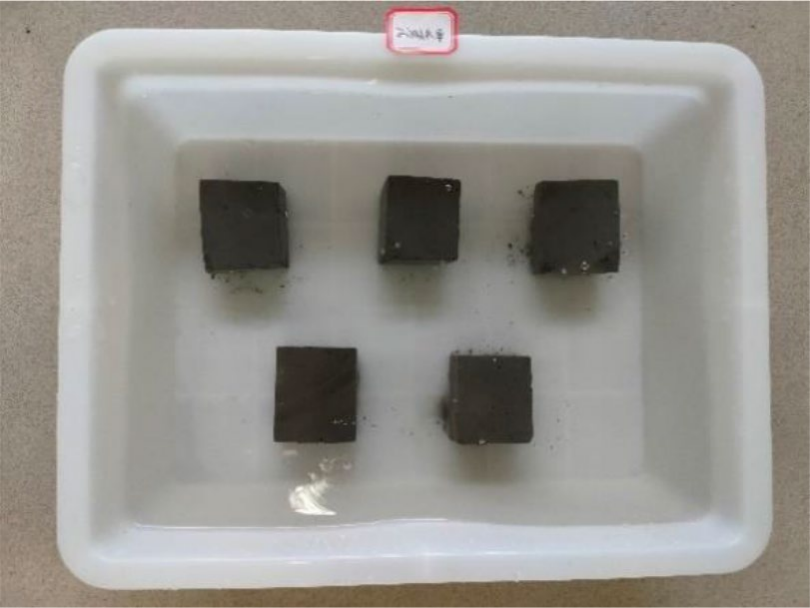
Samples soaking for 24 h.

**Figure 8:**
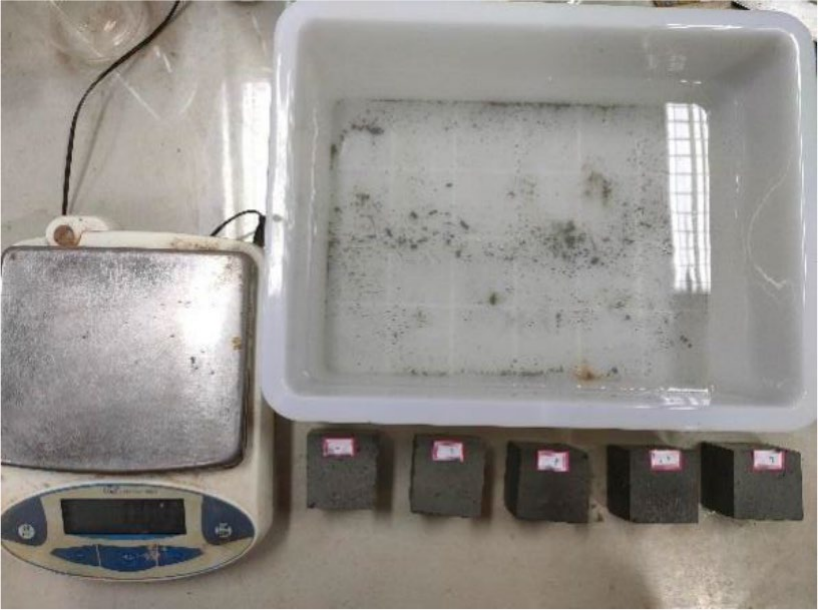
Weighing of test samples.

According to the specifications in “Test Methods for Wall Bricks” GB/T 2542-2012, we took five pieces of 50 mm × 50 mm × 50 mm cube samples, cleaned the samples’ surface with a brush, and numbered them; the numbers were A_1_–A_5,_ respectively. First, we weighed the mass *m*_0_ of each sample in the natural state. Next, placed the sample in a blast drying box set to 105–110 °C to dry to a constant mass *m*_1_ after drying. Next, immersed the dried sample in water at 10–30 °C (as shown in Fig. 7) and took it out after 24 h for repetitive weighing until the difference between three consecutive measurements was less than 0.2% (as shown in Fig. 8), and recorded its mass m_24_.

### Void ratio test

The “hole rate” intuitively reflected the whole situation inside the brick body and indirectly indicated the weathering state of the brick. The void ratio test of the brick samples gave further specific theoretical support for the maintenance and reinforcement of the Kaifeng People’s Conference Hall.

#### Experimental method

Refer to the relevant regulations in "Test Methods for Wall Bricks" (GB/T 2542-2012), we took five pieces of 50 mm × 50 mm × 50 mm cube samples to clean the surface with a brush. Placed a graph paper on the top of the cleaned, cut brick surface and repeatedly rolled a rubber roller on the graph paper so that the cut surface entirely impinged on the graph paper. Noted the holes invaded on the graph paper, the size of the grooves on the cutting surface, and the size of the cut surface on the sample. Any irregular outer edge was analyzed by counting the number of grids to determine the top, bottom, left, and right areas. Pieces that were less than one grid were pieced together to reflect the actual situation of the hole as much as possible.

### Frost test

Groundwater erosion dissolved soluble salts in the wall bricks located close to the ground. As a result, brick materials displayed a porous microstructure with criss-cross micropores^38^. Micro-pores form channels enabling water transfer and storage, and at the same time, drove salt migration. With the moisture evaporation from the brick, soluble salt precipitation occurred on the surface of the brick. The dissolution and crystal expansion of a large amount of soluble salt caused the surface of the brick masonry to be powdered and peeled, which increased the internal pores of the brick and weakened its frost resistance^39–42^, thus necessitated the frost testing of the sample. Through the frosting situation of the sample, the influence of the local conditions in Kaifeng on the sample was evaluated, providing a reference index for the repair of the Kaifeng People’s Conference Hall.

#### Experimental method

According to "Test Method for Wall Bricks" (GB/T 2542-2012), we took five pieces of 50 mm × 50 mm × 50 mm cube samples to clean the surface with a brush and number them. Placed the sample in a blast drying oven at 100–110 °C for 24 h and then took it out. After the sample was removed, cooled to room temperature, the top surface of the sample or the side with holes facing up on a shallow pan filled with distilled water, and then covered the shallow pan with a transparent cling film. Thus, part of the sample was exposed to the air. The sample was thus immersed in the pan for seven days. Water was added frequently in the first two days of the test to maintain the height of the water surface in the shallow pan. And we only needed to soak the sample in water after two days. During the testing, the ambient temperature was 16–32 °C, and the relative humidity was 35–60%. After 7 days of testing, the sample was removed from the water and placed in the same environmental conditions for 4 days. After 4 days, the sample was placed in a blast drying oven at 100–110 °C and dried to constant mass. After drying, the sample was taken out, cooled to room temperature, and recorded the sample's degree of frosting.

### Test of compressive strength

The maximum axial stress that the specimen withstood without lateral pressure confinement is called the unconfined compressive strength, which accurately reflected the physical and mechanical properties of the sample^43^. Therefore, measuring the compressive strength of bricks was used to assess the strength of bricks.

#### Experimental method

Placed the prepared cube sample flat in the center of the pressure plate and loaded it perpendicularly to the pressure surface. The load had to be uniform, stable and void of any impact or vibration. Prepared the test sample first and performed physical centering adjustment and compression stability adjustment during preloading to ensure that the deformation difference between the two sides of the test piece was less than 15%; otherwise, it needed to be reloaded again. After preloading was satisfactorily complete, we set the parameters of the machine. Due to the reason for the instrument itself, only force control will affect the test curve. Hence the loading method was divided into a two-stage control. The first stage used 5 kN/s force control, and the second stage used 1 mm/min axial displacement control. The cube sample cracked slowly during the compression process, and the surface peeled off slightly. When the test sample had a relatively large failure deformation, any further loafing was stopped until the test sample was destroyed and the maximum load was recorded.

### Softening coefficient test

The softening coefficient characterized the material's water resistance and was used as the basis for selecting the material. The softening coefficient value was generally between 0 and 1. Typically, those with a softening coefficient greater than 0.85 were considered water-resistant materials and vice-versa when the softening coefficient was less than 0.85. Therefore, materials with a softening coefficient greater than 0.85 must be selected for essential buildings or structures in water or humid environments for a long time. For materials lightly exposed to moisture or secondary structures, the softening coefficient should not be less than 0.70. A softening coefficient test done on the sample assessed the water-resistance.

#### Experimental method

**Figure 9:**
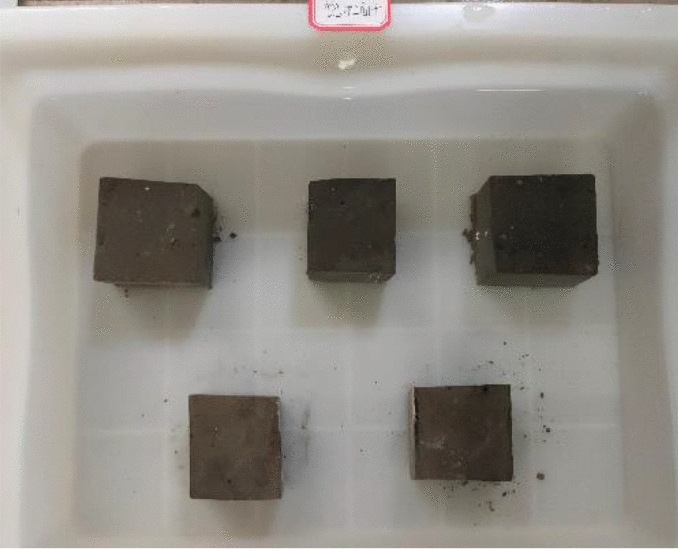
State of dry saturated samples.

**Figure 10:**
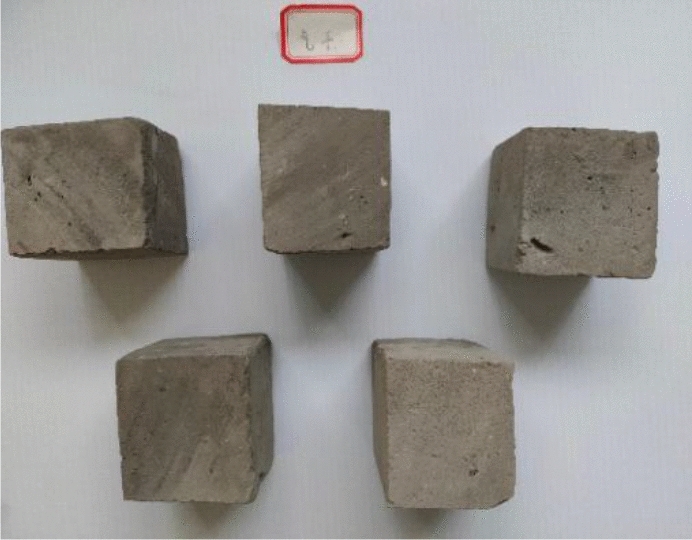
State of air-dried samples.

Ten 50 mm × 50 mm × 50 mm cube samples were taken; five samples were used for the strength test in a saturated dry state, and the other five were used for the strength test in the air-dry state. First, five samples were immersed in water (at 15–25 °C); the water surface was maintained 20 mm higher than the sample surface, and took the samples out after 4 days of soaking. Next, placed the removed samples on a wire mesh rack to drain the free water for 1 min, and then wiped off the excess water on the sample surface with a damp cloth. At this time, the samples were saturated but dry (as shown in Fig. 9). Next, put the other five samples in a non-ventilated room set at a temperature of less than 10 °C for 72 h; the samples were air-dried (as seen in Fig. 10). Finally, the saturated dry and air-dried samples were tested using the aforementioned compressive strength test. The compressive strength ratio in the dry form of the saturated sample to the strength in the air-dry state was the value determined as the softening coefficient.

## Results and discussions

### Phase analysis

**Figure 11:**
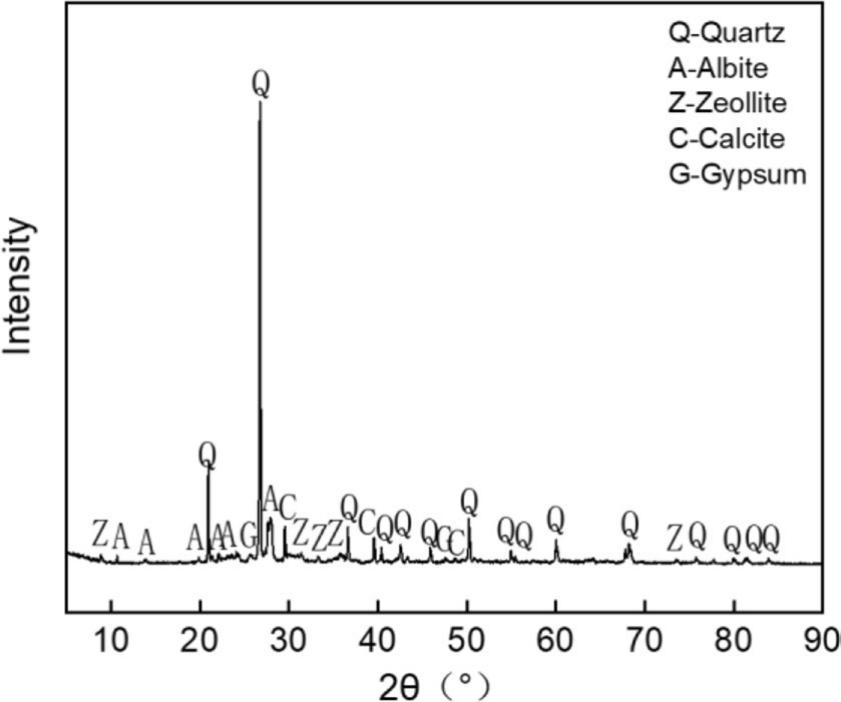
XRD pattern of ordinary sintered red brick.

**Figure 12:**
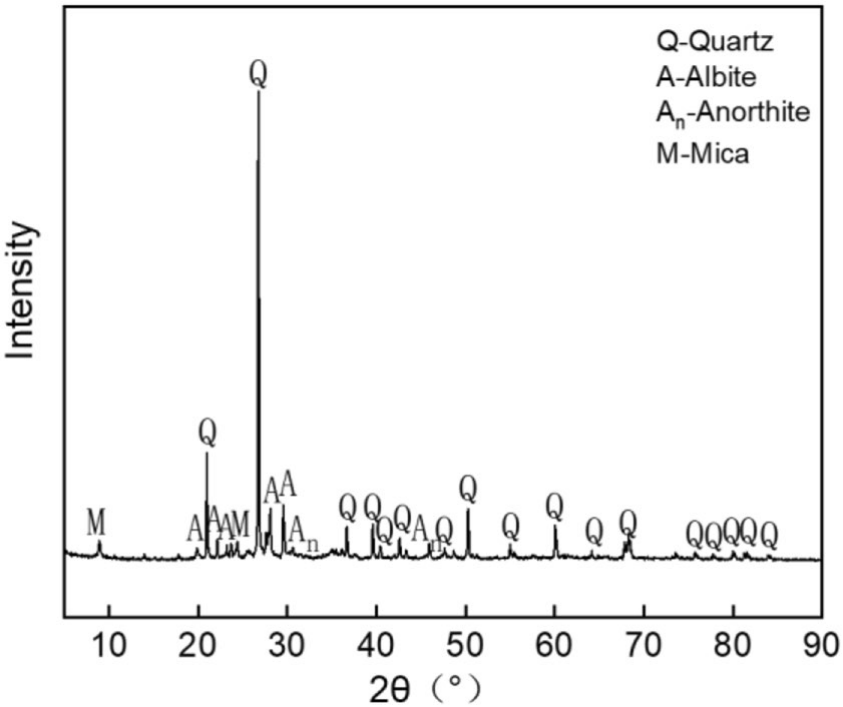
XRD pattern of clay blue brick sample.

We combined the drawing tools to draw the mineral composition diagram (as shown in Figs. 11, 12). Figure 11 indicated that the main mineral composition of ordinary sintered red bricks was quartz, but it also contained albite, zeolite, calcite, and gypsum. Similarly, the annotated Fig. 12 (for blue clay brick) illustrates that quartz's diffraction peaks were 20.8°, 26.6°, 36.5°, 39.4°, 40.3°, 42.4°, 47.5°, 50.1°, 55.3°, 60.9°, 64.0°, 68.1°, 68.3°, 73.5°, 77.6°, 81.2°, 81.5°, and 84.972°. The diffraction peaks for albite were 22.1°, 23.5°, 27.9°, 28.7°, and 29.4°. The diffraction peaks for anorthite were 31.2° and 45.4°. The diffraction peaks for mica were 8.8° and 24.1°. Hence, the main mineral composition of the blue clay brick was also quartz. In addition to quartz, it also contained albite, mica, and anorthite. There were no high-temperature sintered products of mullite and cristobalite; hence the sintering temperature did not exceed 1000 °C^44–51^.

### Characteristics of pore

**Figure 13:**
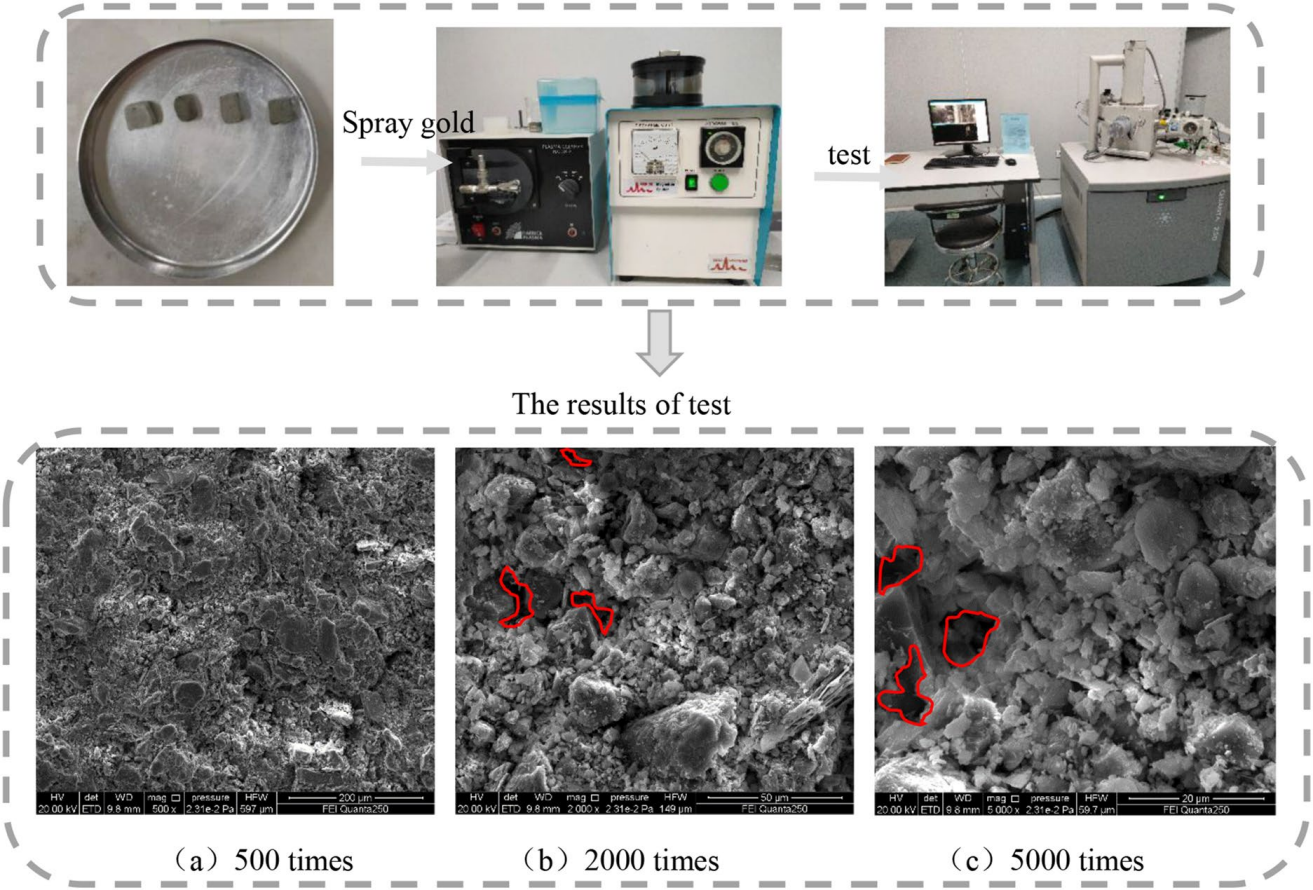
Specimen electron microscopy results.

The pore characteristics of the sample are seen as electron micrographs in Fig. 13, at increasing magnifications of 500×, 2000×, and 5000×. These magnified images of the blue clay sample show that the internal pores have prominent characteristics, mainly the particle structures, and the pores between the structures are significant, as shown by the red outlines. In addition, we observed many fine holes between the particles in the brick body, which are the main internal factors that cause the poor frost resistance and sulfate erosion resistance of the blue bricks. At the same time, it was noted that the internal particles of the sample were of varying sizes, with fewer large particles, significantly more small particles, and higher density. Therefore, based on the above analysis, we saw evidence that the blue clay bricks of the Kaifeng People’s Conference Hall wall were of high quality.

### Density, moisture content, and water absorption test results and analysis

The density of the sample is calculated as follows:

1 $${p}_{0}=\frac{{m}_{1}}{L\times B\times H}.$$

The moisture content of the sample is calculated by the following formula, accurate to 0.1%:

2 $${W}_{1}=\frac{{m}_{0}-{m}_{1}}{{m}_{1}}\times 100.$$

The water absorption of the sample soaked in room temperature water for 24 h is calculated by the following formula, accurate to 0.1%:

3 $${W}_{24}=\frac{{m}_{24}-{m}_{1}}{{m}_{1}}\times 100,$$

where L is the sample length in cm, B is the sample width in cm, H is the sample height in cm, p_0_ is the apparent density, accurate to 0.1 g/cm^3^, m_0_ is the sample quality in g, m_1_ is the sample dry mass in g, m_24_ is the wet mass of the sample after being immersed in water for 24 h in g, W_1_ is the moisture content of the sample under normal conditions, W_24_ is the water absorption of the sample soaked in water at room temperature for 24 h.

**Figure 14:**
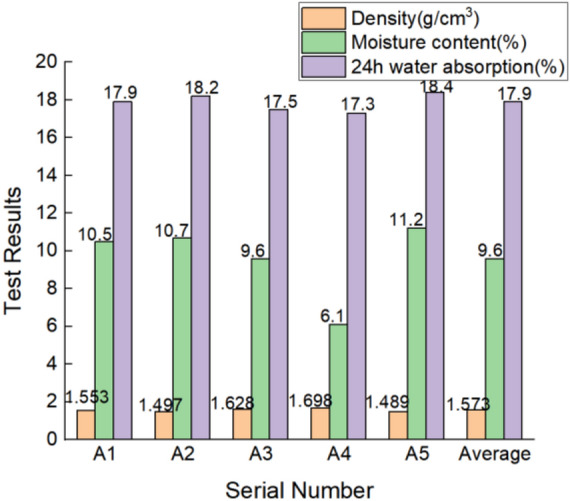
Bar charts of density, moisture content, and water absorption of each sample.

The calculated results for each sample’s density, moisture content, and 24 h water absorption are shown in the bar charts in Fig. 14; A1–A5 are the five sample numbers.

The test results in Fig. 14 show that the density for the five samples ranged from 1.489 to 1.698 g/cm^3^, with an average value of 1.573 g/cm^3^. The density of the five samples and the average result was lower than the density value of 1.70 g/cm^3^ of standard clay bricks. Under the natural environmental forces on the wall for nearly a 100 years, affected by weathering, there were many pores in the brick body so that the compactness of the sample had slightly reduced. The water content of the five samples was 6.1–10.7%, and the water absorption was 17.3–18.4%. The water absorption of five samples was partially lower than the average value of “Sintered Ordinary Brick” (GB/T 5101-2017) (18%), but some of them reached more than 18%, with an average value of 17.9%. Although it was close to the average value (18%) of “Sintered Ordinary Brick” (GB/T 5101-2017), the water absorption was slightly higher. In addition, from the bar charts, it was seen that the higher the density for the same sample, the lower the water content and water absorption, which reflected that the denser the material and the smaller the internal porosity of the material.

The density, moisture content, and water absorption depended on the brick’s size and the number of pores. The number and size of the internal pores are some of the main factors that affect the durability of the wall that cause the wall deterioration. Kaifeng is rainy in summer, and the water absorption of the blue bricks on the wall is relatively large. After the wall bricks were saturated with water, they become easily affected by temperature^52–54^. Due to thermal expansion and cold contraction at high temperatures, the blue clay brick will crack and peel off the wall surface. For clay bricks with low density, high water absorption, and high porosity, a colorless, transparent, and well-permeable organic silicon reinforcement can be selected for repair and reinforcement^55^.

### Void ratio test results and analysis

The void ratio of the sample is calculated as follows:

4 $$H=\frac{{S}_{1+}{S}_{2}}{{S}_{0}}\times 100,$$

where H is the void ratio rate, accurate to 0.1%, S_1_ is the sum of the area of the torn hole in mm^2^, S_2_ is the sum of the groove area of the cross-section in mm^2^, S_0_ is the cross-sectional area of the test piece in mm^2^.

**Figure 15:**
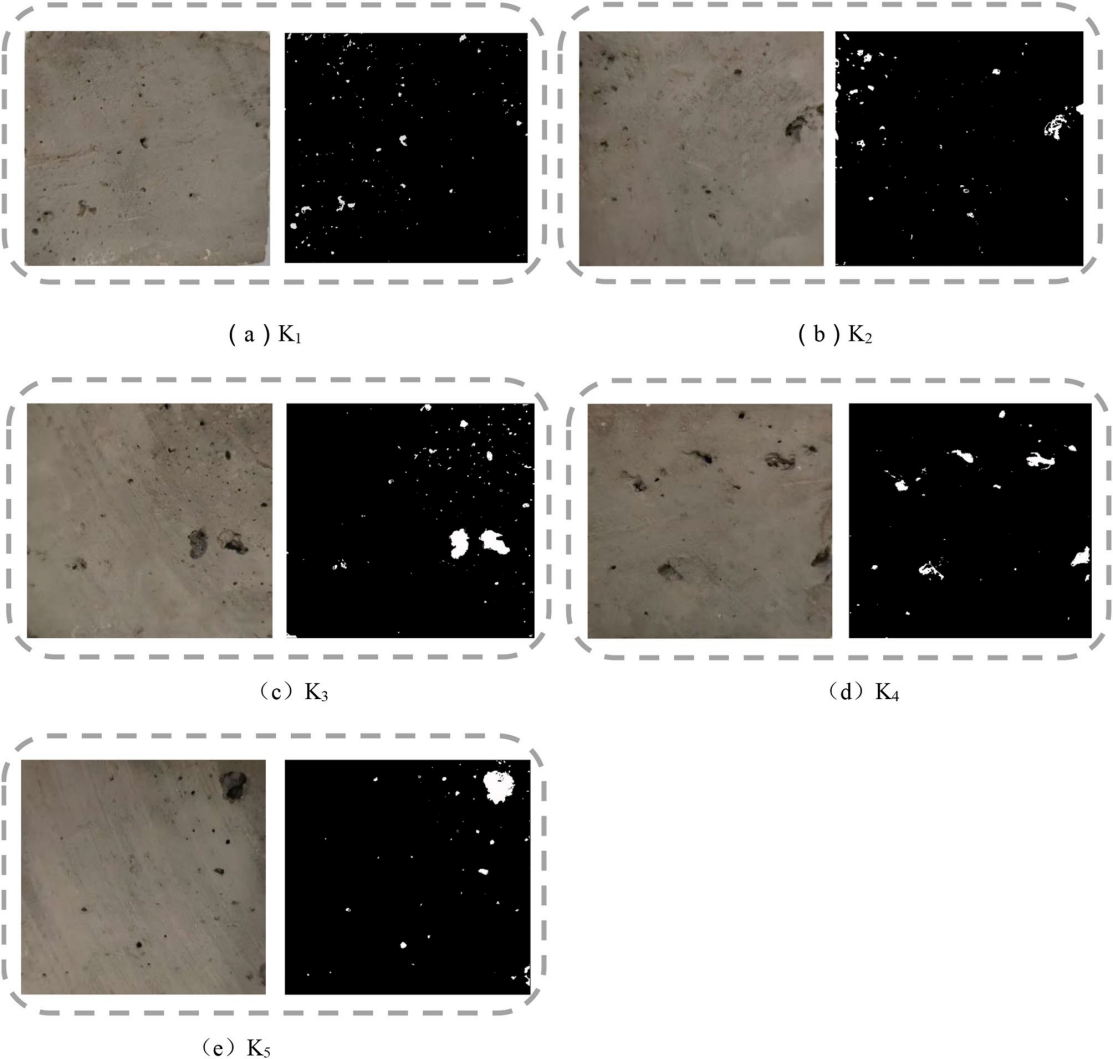
Hole inside the sample.

**Figure 16:**
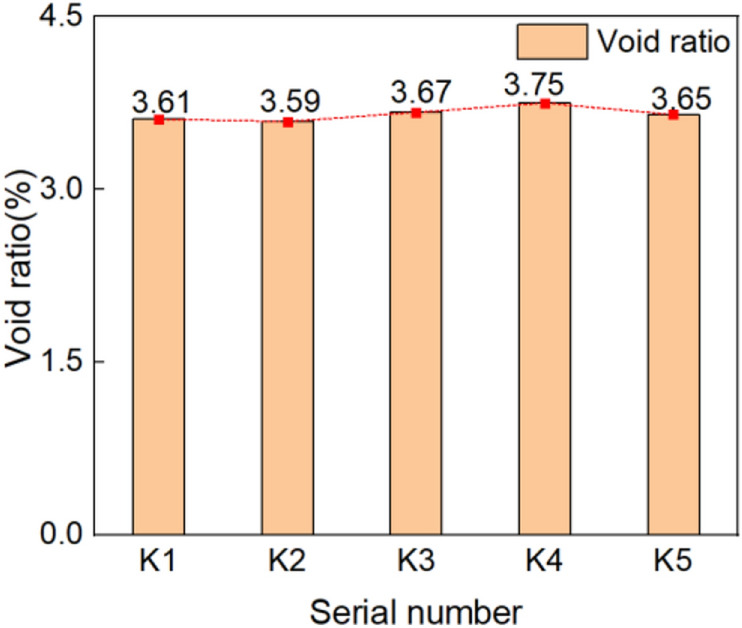
Void ratio histogram.

The measured results were summarized (as shown in Table1). In addition, the internal pore diagram of the sample (as shown in Fig. 15) are respectively the samples of K_1_–K_5_. The calculated result of the void ratio for each sample was represented by a bar chart (as shown in Fig. 16).

**Table 1:** Void ratio measurement result.

Serial number	K_1_	K_2_	K_3_	K_4_	K_5_
S_1_ + S_2_ (mm^2^)	90.254	89.673	91.736	93.639	91.255
S_0_ (mm^2^)	2500	2500	2500	2500	2500
H (%)	3.61	3.59	3.67	3.75	3.65
Average value (%)	3.65				

K_1_–K_5_ are the numbers of five void ratio test samples.

Due to the limitations of the test instrument, we used the vernier caliper to measure the size of hole. Accurate to mm. The results in Table 1 showed that the void ratio of the brick sample lay between 3.59 and 3.75%, and lower than the void ratio of standard solid clay bricks (25%). In conjunction with the local Kaifeng environmental conditions and with the long-term groundwater migration, salt erosion, freeze–thaw cycles, and alternating cold and heat, the blue clay bricks of the Kaifeng People’s Conference Hall produced internal pores that were invisible to the naked eye^56–60^. This kind of blue brick with relatively large sand content is easily affected by the environment. Located beside the Yellow River, Kaifeng is cold in winter and rain in summer, so its cultural relics are more vulnerable to damage caused by freeze–thaw cycles. In the process of repeated freezing and melting, the phase state of the water between the pores of the blue bricks in the wall constantly changes, resulting in the internal pores being constantly in microscopic expansion force, and finally leading to the expansion of the pore structure. The expansion of internal pores not only leads to the decrease of the density of blue bricks on the wall, but also increases the water absorption, and slowly expands the hole visible to the naked eye, so that the number of brick surface holes in the original bricks have caused holes.

The internal pores led to a density decrease in the blue clay bricks, increased water absorption, and slowly expanded into holes visible to the naked eye. The gradual increase in the number of holes and the gradual enlargement of the holes accelerated the weathering of the wall, causing more severe deficiencies, such as flaking, cracks, and salt panning on the wall. Although the void ratio of the blue clay bricks on the wall was small, we could not ignore it. The quantitative change caused qualitative changes. The weathering and loss of the blue bricks inevitably caused the weakening and instability of the entire structure. Therefore, appropriate materials needed to be adopted in time, and reasonable measures were taken to reinforce the walls. The reinforcement must meet the principle of “replace the old for new” in restoring historical buildings^61,62^_,_ and the original building layout and style should not be changed or damaged.

The first picture shows the surface of the brick sample in Fig. 15a–e. In order to make readers see the holes on the surface of the brick more clearly, the second picture Outlines the surface of the brick, highlighting the position and shape of the holes in white.

### Frost test results and analysis

**Figure 17:**
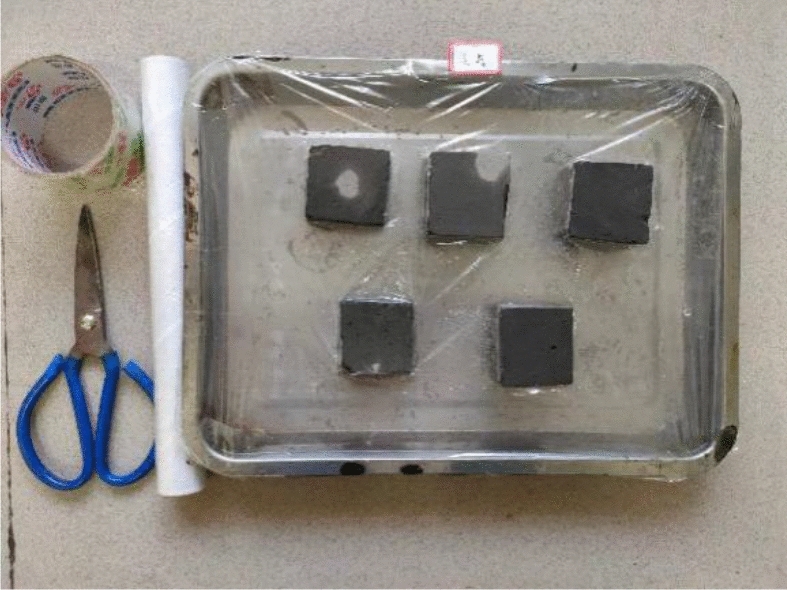
Frost testing process.

**Figure 18:**
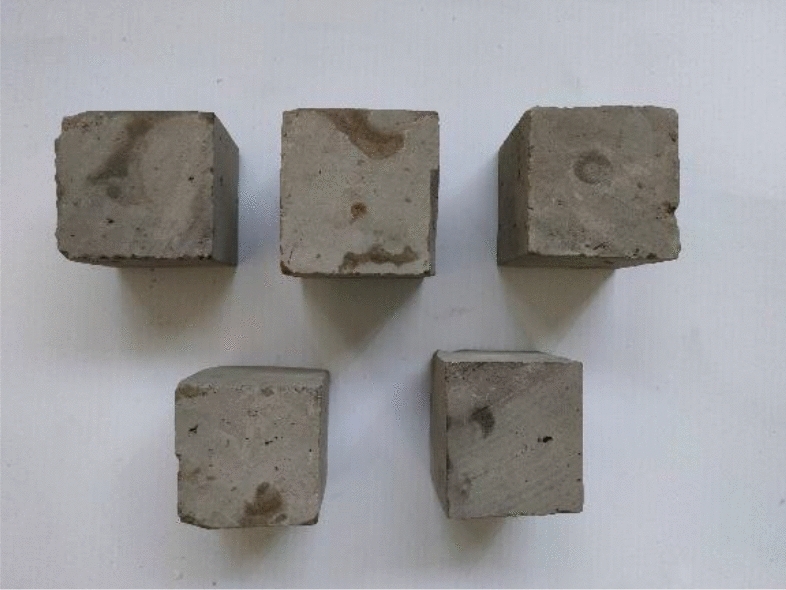
Evidence of sample frosting.

The frosting test process and test samples are seen in Figs. 17 and 18. According to the “Test Method for Wall Bricks” (GB/T2542-2012), the evaluation standard of the degree of frosting, combined with the test results, we noticed small but yet noticeable frost film appeared on the sample surface, but the surface of the sample was still seen; hence the sample frosting was assessed as ‘slightly frosted’.

The degree of frosting of the blue clay bricks was related to the internal material composition and pore characteristics, and also the groundwater migration with the dissolution of salt also playing a key role^63–65^. Due to the brick’s porous nature, surface water and groundwater permeate the wall through the capillary pores, simultaneously promoted salt migration. The salt dissolved in the brick wall penetrated the wall appearing after the water evaporated. The apparent salt would accelerate the powdering of the wall on interacting with moist air. Under long-term environmental effects, the chalking intensified to encroach into the wall's interior. If these continue, it will cause foundation instability. In addition, the Kaifeng soil is relatively alkaline, and the groundwater level is relatively high. Based on the experimental results, the influence of the local conditions in Kaifeng on the brick walls should not be ignored. Given this situation, traditional desalination must be encouraged. The soluble salt on the wall surface was washable with distilled water and removed, and the insoluble salt was removed by scrubbing with 2% dilute hydrochloric acid. This was very necessary for the scientific protection of the people’s meeting place.

### Compressive strength test results and analysis

#### Experimental phenomenon

**Figure 19:**
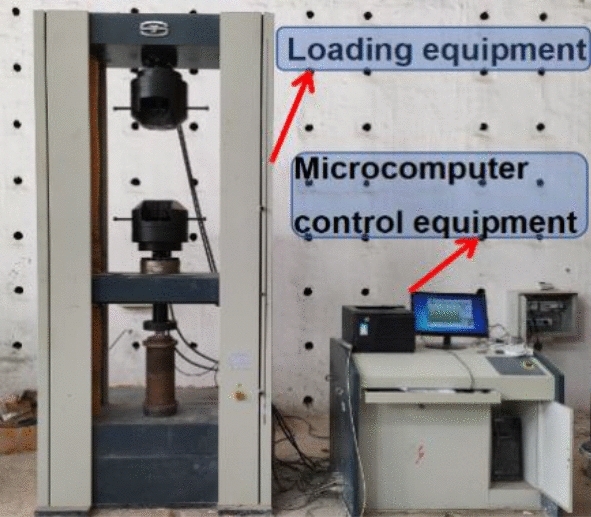
Microcomputer controlled testing machine.

**Figure 20:**
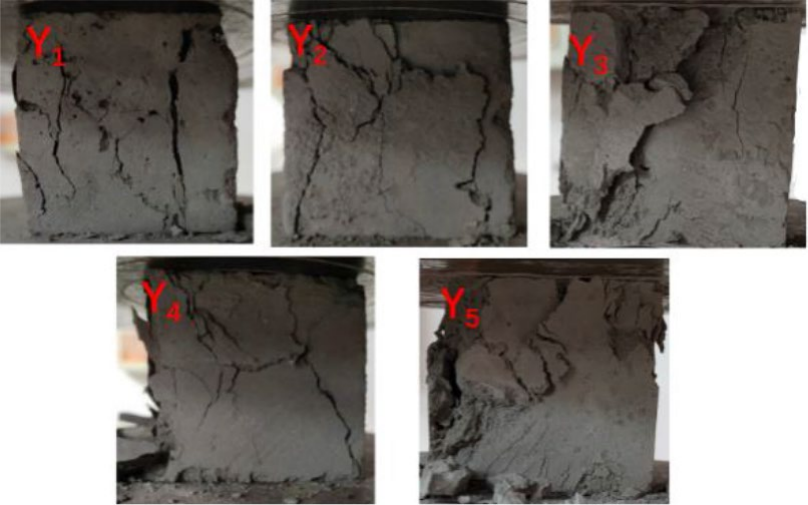
The sample failure forms at ultimate load.

At the commencement of loading, slight cosmetic cracking appeared at the edges and corners of the test samples. The initial crack direction was mainly vertical or slightly oblique. With the continued increase of the vertical load, the internal stress in the sample concurrently increased, the width of the micro-cracks formed at the initial stage of loading increased, the cracks continued to extend along the direction of the initial cracks, and simultaneously new cracks were gradually formed. When the load was approaching the ultimate load, the vertical surfaces of the specimen began to fall off locally. When the load reached the limit load, the crack width of the sample further increased, the sample’s surface is locally peeled off, the load-bearing capacity dropped rapidly, and the sample failed. The test equipment is seen in Figs. 19 and 20. During the loading process, the sound of the specimen being crushed was audible.

#### Test results and analysis

Calculate the compressive strength values of five specimens according to the following formula, accurate to 0.01 MPa:

5 $${f}_{c}=\frac{F}{LB}.$$

The coefficient of variation is introduced to analyze the discrete type of the sample. The standard deviation s and the coefficient of variation δ of the sample are calculated as follows:

6 $$\mathrm{s}=\sqrt{\frac{1}{n-1}\sum_{i=1}^{n}{\left({f}_{i-}\overline{f }\right)}^{2}},$$

7 $$\updelta =\frac{s}{\overline{f} },$$

where ${\mathrm{f}}_{\mathrm{c}}$ is the compressive strength in MPa, $\mathrm{F}$ is the maximum failure load in N, $\mathrm{L}$ is the length of the pressure surface in mm, $\mathrm{B}$ is the width of the pressure surface in mm, $\mathrm{S}$ is the standard deviation, δ is the coefficient of variation, $\overline{\mathrm{f} }$ is the average value of sample strength in MPa, ${\mathrm{f}}_{\mathrm{i}}$ is the strength of a single sample in MPa.

**Figure 21:**
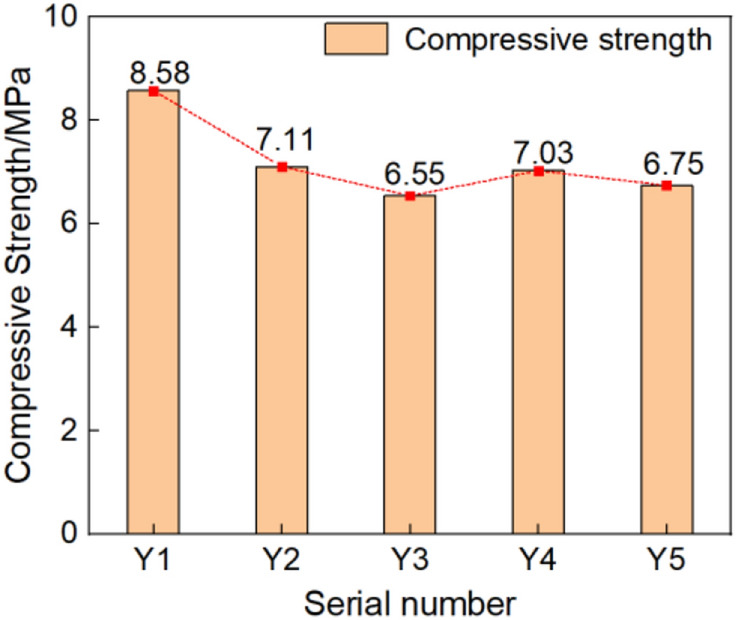
Bar chart of compressive strength of each sample.

**Figure 22:**
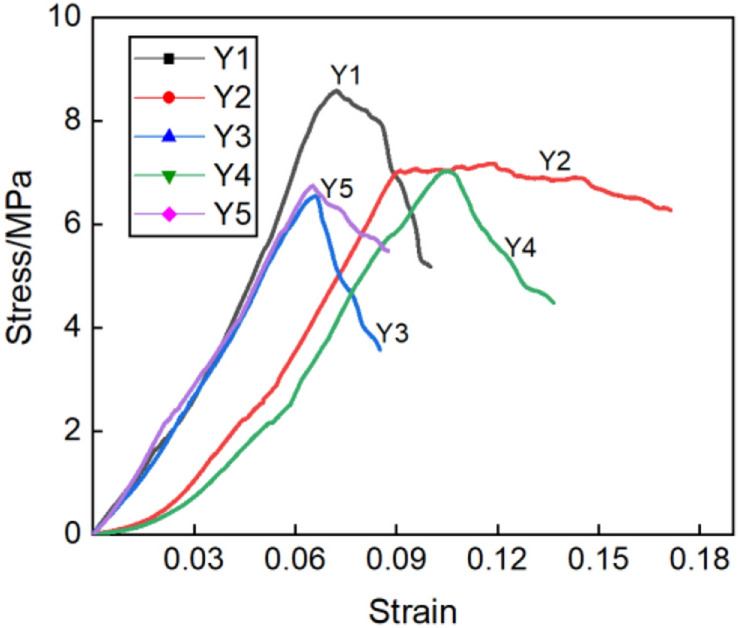
Stress–strain curves of test samples.

The compression test results are in Table 2 and further illustrated as a bar chart in Fig. 21. The stress *σ* of blue brick material was the load value divided by the cross section area of the sample. That's the forced per unit area. The strain *ε* of blue brick material was obtained by passing a strain gauge on the side of blue brick. The strain gauge was connected to the strain tested by a half-bridge connection, and the micro-deformation can be obtained through the signal acquisition and data processing of the strain tested. The specific method was that the material strain gauge was respectively arranged at the lateral center point of the uniaxial compression sample. In the process of sample loading test, the relationship between the force and deformation of the brick material was obtained by real-time monitoring. The curve of specific test results is shown in Fig. 22.

**Table 2:** Compression test results.

Serial number	Y_1_	Y_2_	Y_3_	Y_4_	Y_5_
Failure load (kN)	21.45	17.79	16.36	17.57	16.87
Pressure surface area (mm^2^)	2500	2500	2500	2500	2500
Compressive strength (MPa)	8.58	7.11	6.55	7.03	6.75
Average (MPa)	7.204				
Standard deviation	0.64				
Coefficient of Variation	0.09				

Y_1_–Y_5_ are the numbers of five compressive strength test samples.

Using the slope of the two points on the graph corresponding to 0.6 times the ultimate strength and 0.3 times the ultimate strength in the stress–strain curve, the elastic modulus of the sample was calculated as 789.8 MPa. Test results in Fig. 21 show that the strength grade of the blue clay brick was less than MU10 for standard sintered brick, and the strength did not meet the relevant requirements specified in the current masonry code, nor did it meet the criteria in the masonry seismic code. The current compressive strength value did not indicate the initial mechanical performance indicators of the blue clay brick. The brick-making technology of a hundred years ago was known to be exquisite, but the blue clay brick tested was severely weathered through the long-term environmental effects. Long-term weathering had caused the mechanical properties of ancient bricks to deteriorate. According to research experience, a high-molecular polymer surface repair agent would be appropriate to repair the blue clay bricks of the wall. Referring to other scholars’ observations on the compressive strength of standard sintered bricks, the coefficient of variation of compressive strength of standard sintered bricks was between 0.07 and 0.13, and the coefficient of variation of compressive strength of concrete hollow block masonry reported by Zheng et al.^66^ was between 0.09 and 0.16. Compared with other bricks or blocks, the coefficient of variation of this blue clay brick sample is less. Hence the discrete type of the brick sample is smaller, and the uniformity was better.

### Softening coefficient test results and analysis

**Figure 23:**
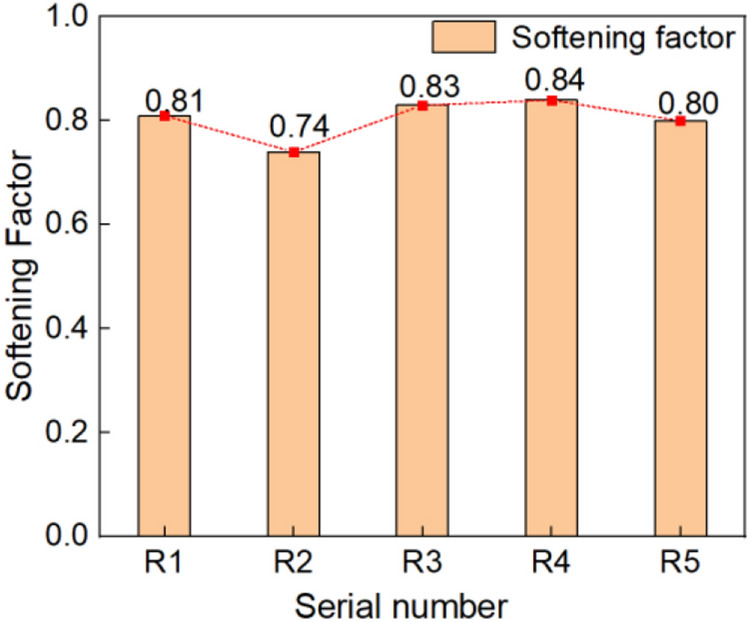
Histogram of softening coefficient of each sample.

The results of the softening coefficient of the blue clay brick samples are given in Table 3. The results of the softening coefficient of each test sample were illustrated as a bar chart in Fig. 23. It was evident from the test results that the average softening coefficient of the blue clay brick sample was 0.80, and results ranged between 0.70 and 0.85. Therefore, the water-resistance of the blue clay brick was good. Kaifeng is located in the Central Plains, with rainy summers and cold winters. After nearly a hundred years of wind and rain erosion, the water-resistance of the blue clay brick had remained good. Although the current test results could not accurately verify the water-resistance of the original blue clay brick, it was comforting to know that the blue clay brick maintained a high sintering quality.

**Table 3:** The results of softening coefficient.

Serial number	R_1_	R_2_	R_3_	R_4_	R_5_
Dry compressive strength of saturated surface (MPa)	3.88	4.66	4.52	3.17	3.04
Air-dry pressure (MPa)	4.81	6.27	5.47	3.79	3.80
Softening coefficient	0.81	0.74	0.83	0.84	0.80
Average	0.80				

R_1_–R_5_ are the numbers of the results that the ratio of the compressive strength of the five samples in the saturated dry state to the compressive strength of the five samples in the air-dry state.

### The difference between modern blue bricks and red bricks and antique-style bricks

The firing process of blue brick was complex, the cost was high and the output was small, it was difficult to realize automation and mechanization production, and most of it was made by pure hand. Accordingly, the intensity of blue brick was far greater than red brick. Blue brick had high density, durability and strong resistance to weathering. The “Qin (blue) bricks and Han tiles” of ancient China have been well preserved for thousands of years. This was the best proof of excellent performance of blue brick. The mineral composition of red bricks and blue bricks was different, but the main component was quartz. Density, moisture content, and water absorption test results showed that the density for the five samples ranged from 1.489 to 1.698 g/cm^3^, with an average value of 1.573 g/cm^3^. The density of the five samples and the average result was lower than the density value of 1.70 g/cm^3^ of standard red bricks. The water content of the five samples was 6.1–10.7%, and the water absorption was 17.3–18.4%. The water absorption of five samples was partially lower than the average value of “Sintered Ordinary Brick” (GB/T 5101-2017) (18%), but some of them reached more than 18%, with an average value of 17.9%. Although it was close to the average value (18%) of “Sintered Ordinary Brick” (GB/T 5101-2017), the water absorption was slightly higher. Void ratio test results showed that the void ratio of the brick sample lay between 3.59% and 3.75%, and lower than the void ratio of standard solid clay bricks (25%). Compressive strength test results showed that the strength grade of the blue clay brick was less than MU10 for standard sintered brick, and the strength did not meet the relevant requirements specified in the current masonry code, nor did it meet the criteria in the masonry seismic code. It can be seen from the test results that the properties of the modern brick have decreased under the influence of weathering for nearly a hundred years.

During the renovation of ancient buildings, it was found that the quality of modern-fired antique-style bricks was significantly poorer than that of modern bricks. Many ancient masonry buildings with blue bricks have been preserved for hundreds of years, while the antique-style bricks produced in modern times will soon be age. Hence, the ancient buildings cannot be effectively renovated and preserved with modern-fired bricks. Therefore, it was very necessary and meaningful to carry out material experimental research on modern and traditional architectural blue brick materials and scientifically evaluate their properties. Through test results, we gathered the needed basic properties. When repairing a building structure from this period, materials needed to be similar to maintain compatibility with the historical building materials and achieve “compatible renovation”.

## Conclusions

1. The main mineral composition of the blue clay brick materials in the People’s Conference Hall walls was quartz, followed by albite, mica, and anorthite. There were no high-temperature sintered products of mullite and cristobalite. Therefore we inferred that the sintering temperature of this batch of bricks did not exceed 1000 °C. It was crucial to pay attention to the preparation of blue clay brick materials with similar material properties.
2. From the scanning electron microscopy images, the pores of the blue clay bricks on the wall have prominent pore characteristics, mainly composed of particle structures, with fewer large particles inside, with more small particles, with higher density. Because the modern masonry bricks were made differently from the current clay bricks, the compactness of the contemporary building clay bricks was relatively high. Therefore, compared with standard sintered bricks, modern clay bricks had a slightly higher weathering resistance.
3. The density of the blue clay bricks on the Kaifeng People’s Conference Hall walls was 1.573 g/cm^3^, which was less than the density of 1.70 g/cm^3^ of standard clay bricks. However, the water absorption of 18% was marginally more significant than that of standard sintered bricks. Therefore, for blue clay bricks with low density, high water absorption, and high porosity, colorless, transparent, and less-viscous organic silicon permeant could be selected for repair and reinforcement.
4. Under the conditions of long-term natural weathering and improper use and transformation by later generations, ancient bricks’ materials and mechanical properties have declined. The compressive strength grade of the People’s Conference Hall blue clay bricks was less than MU10, which does not meet the relevant requirements in the current masonry code, nor does it meet the needs of the masonry seismic code. Through research experience, a high-molecular polymer surface repair agent could be selected to repair the blue clay bricks of the wall.
5. The softening coefficient of the blue wall brick was 0.80, and ranging between 0.70 and 0.85. The “Uniform Technical Code for the Application of Wall Materials” GB 50574-2010 stipulated that products with a softening coefficient of less than 0.85 should not be used in wet parts with a softening coefficient of less than ± 0.000. To prevent structural instability arising from the weak water-resistance of the material, a waterproof coating with better water resistance could be selected for repair in the wet part.
